# COVID-19 pandemic and vaccine hesitancy in a Brazilian state capital

**DOI:** 10.1038/s41598-026-45085-0

**Published:** 2026-03-25

**Authors:** Ana Isabel do Nascimento, Danilo dos Santos Conrado, Lisany Krug Mareto, Micael Viana de Azevedo, João Cesar Pereira da Cunha, Gabriel Serrano Ramires Koch, Laysa Gomes Osório, Samara Tessari Pires, Letícia Suemi Arakaki, Sara Raquel Pinto Borges, Robson França Gomes e Silva, Rodrigo Mayer Pucci, João Guilherme de Novaes Corrêa, João Vitor Barrio, Maria Eduarda de Souza Rodrigues, Márcio José de Medeiros, Ana Paula Sayuri Sato, Maria Elizabeth Araújo Ajalla, Cláudia Du Bocage Santos-Pinto, Everton Falcão de Oliveira

**Affiliations:** 1 Programa de Pós-Graduação em Doenças Infecciosas e Parasitárias, Universidade Federal de Mato Grosso do Sul, Campo Grande, MS Brazil; 2 Faculdade de Medicina, Universidade Federal de Mato Grosso do Sul, Campo Grande, MS Brazil; 3 Instituto Politécnico, Universidade Federal do Rio de Janeiro, Macaé, RJ Brazil; 4 Faculdade de Saúde Pública, Universidade de São Paulo, São Paulo, SP Brazil

**Keywords:** Vaccine hesitancy, Coronavirus disease 2019, Perception, Vaccine Hesitancy Scale, Infodemic, Health policy, Epidemiology

## Abstract

The COVID-19 pandemic was disclosed to have both positive and negative impacts on vaccine acceptance. To further comprehend its influences on the population’s perceptions about vaccination, we conducted a cross-sectional study that aimed to evaluate the trends and determinants of vaccine hesitancy before and after the onset of the COVID-19 pandemic, in Campo Grande, a state capital in Brazil. Data was collected through household interviews conducted between 2022 and 2023, using questions addressing both the pre- and post-pandemic periods. Information on socioeconomic and demographic characteristics, COVID-19 vaccine hesitancy, and the number of COVID-19 vaccine doses received was collected. Vaccine hesitancy over time was assessed using the World Health Organization 10-item Vaccine Hesitancy Scale (VHS), applied retrospectively to the periods before and after the beginning of the pandemic. Positive items were reverse-scored so that higher score indicated greater vaccine hesitancy. Some items of the VHS were aligned in two factors (lack of trust and risk perception) and two bivariable and two linear regression models were performed to describe the association between the variables and those factors. We observed an increase in both factors and in the overall VHS score after the onset of the pandemic. Lower trust was associated with older age, whereas lower COVID-19 vaccine hesitancy was associated with a higher number of COVID-19 vaccine doses received. Increased risk perception was associated with higher COVID-19 vaccine hesitancy. These findings suggest that the COVID-19 pandemic contributed to an overall rise in vaccine hesitancy in this population.

**Supplementary Information:**

The online version contains supplementary material available at 10.1038/s41598-026-45085-0.

## Introduction

In Brazil, the COVID-19 pandemic was impactful in several dimensions^1,2^. From February 2020 to April 2022, the country faced a public health emergency which ended in an ongoing discussion about the transition to an endemic scenario^3,4^. This unprecedented historical event was noted to influence vaccine acceptance and overall vaccination rates either positively or negatively^5,6^. As observed in past nationwide health emergencies, vaccine hesitancy has historically posed challenges to disease control, as exemplified by the Vaccine Revolt in Brazil in the early twentieth century (1904), which emerged during the implementation of compulsory smallpox vaccination^7^.

Vaccine hesitancy has been defined as “a motivational state characterized by conflict or opposition toward getting vaccinated”, including intentions and willingness to receive vaccination^8^. Vaccine-hesitant individuals exist along a spectrum, ranging from total refusal to acceptance of vaccines^9^. It is a volatile, multifactorial, and complex phenomenon, influenced by the time and place of its occurrence, as well as by fluctuations in these factors over time^10^.

During the COVID-19 pandemic, vaccine hesitancy became a global concern^11^. Among the numerous factors influencing vaccination acceptance during a pandemic^12^, many were reported during the COVID-19 crisis. However, vaccination acceptance rates in Brazil were reported to be high^13^. Despite the population’s apparent positive reception to the new vaccine, the vaccination process in Brazil was described as slow and challenging^14^.

As vaccine hesitancy may be influenced by multiple factors, including contextual influences (such as historical events, influential leaders, religion, culture), group and individual influences (personal, community, and family experiences), and vaccine-related aspects, such as vaccine scheme, and trust in vaccination services and policies^15^, the comprehension of this phenomenon requires the consideration of both, personal and contextual elements^10^. Over the years, after its conceptualization, several theoretical conceptual models were developed to describe the dimensions of vaccine hesitancy. Among those, the 3 C conceptual model presented three dimensions to unfold this phenomenon: complacency (low perceived risk of diseases driving low perceived need for vaccines), convenience (access to vaccination and willingness to overcome barriers to access vaccines), and confidence (the perception of the health system, policy makers, and vaccine credibility and safety)^16^.

Prior to the COVID-19 pandemic, the 3 C model of vaccine hesitancy was mainly used to classify reasons for vaccine hesitancy rather than to measure its dimensions^17,18^. Issues related to confidence and complacency were described and discussed, both with low prevalence, while convenience-related reasons were also reported^17,18^.

Considering this context, we hypothesize that the COVID-19 pandemic may have enhanced vaccine hesitancy among Brazilian residents and decreased population trust in routine vaccines. To further investigate the potential long-lasting impacts of the pandemic on population acceptance of old and new vaccines, it is crucial to examine their perception toward vaccination over time. Therefore, the study aims to measure trends in vaccine hesitancy before and after the pandemic, in the urban population of the municipality of Campo Grande, Mato Grosso do Sul, Brazil.

## Methods

### Study design and period

This cross-sectional study was nested within a population-based household survey designed to estimate the overall vaccine coverage in Campo Grande. During household visits for vaccination data collection (September 2022–October 2023), participants were also invited to completed a WHO-adapted questionnaire on vaccine hesitancy.

### Sampling

We conducted a stratified two-stage cluster sampling design, following the WHO guidelines^19^. Based on a projected average vaccination coverage of 90% in Campo Grande, with a desired 8% margin of error and a 5% alpha level, a total sample size of 101 was calculated assuming simple random sampling. A pilot study indicated an average of 10 respondents per cluster within a 3-hour interval using a field team of 6 researchers. Considering an intracluster correlation of 0.33, the design effect was set to 3. Using the WHO’s method, the estimated number of clusters was 30.3, rounded up to 30. Clusters were selected through simple random sampling without replacement using the 2021 IBGE (Brazilian Institute of Geography and Statistics, IBGE) census sector map^20^. The pilot study cluster was included in the final sample.

The number and selection of households were determined based on the pilot study. An average of 1.5 residences were visited to find an eligible participant, with an inflation factor of 1.05 applied to account for refusals and non-respondents. The average number of respondents per day was 10. Therefore, each cluster included 15 randomly selected households. Detailed information on the sampling process is provided in Supplementary Methods. Random sampling and spatial allocation of clusters and households were conducted using the sf package in R 3.4.2.

### Participants and data collection

All residents aged 12 years or older who were present in the households at the time of the survey visit were eligible for inclusion in the study.

The data was collected through face-to-face interviews. We collected socioeconomic and demographic data, including age, education (in years of study), number of residents per household, sex, race/ethnicity (White or Black/Mixed/Indigenous/Asian descent), household income, access to piped water (yes/no), and access to sanitary sewerage (yes/no). Household income was categorized as low and non-low according to the Brazilian Federal Government criteria for eligibility for social benefits^21^. Low-income was defined as a monthly per capita income equal to or less than half of the Brazilian minimum wage, following federal eligibility criteria for social benefits.

Variables related to COVID-19 vaccination included the self-reported number of vaccine doses received (none, 1, 2, or ≥3) and the self-reported COVID-19 vaccine hesitancy (yes/no). At the time of the study, COVID-19 vaccines were already available to the population, and the recommended primary vaccination schedule ranged from one to four doses depending on the manufacturer, alongside ongoing discussions on booster doses^22^. Vaccine hesitancy was assessed after presenting participants with the 2015 conceptual definition of vaccine hesitancy^15^.

Lastly, vaccine hesitancy was assessed using the 10-item Vaccine Hesitancy Scale (VHS) developed by the SAGE Work Group (Strategic Advisory Group of Experts)^23^. The scale was translated into Portuguese and culturally adapted to the study population^17^. It consists 10 statements rated on a 5-point Likert scale (1 = strongly disagree, 2 = disagree, 3 = neither agree nor disagree, 4 = agree, 5 = strongly agree). The VHS were administered twice to each participant: first referring to the period before the onset of the COVID-19 pandemic, and then referring to the current period after the start of the pandemic. All items were answered considering vaccination in general, including COVID-19 vaccines. The VHS items are:

1. Vaccines are important for my health.
2. Vaccines are effective.
3. Having myself vaccinated is important for the health of others in my community.
4. All vaccines offered by the SUS (Unified Brazilian Health System) in my community are beneficial.
5. New vaccines carry more risks than older vaccines.
6. The information I receive about vaccines from the vaccine program is reliable and trustworthy.
7. Getting vaccinated is a good way to protect myself from disease.
8. Generally, I do what my doctor or health care provider recommends about vaccines.
9. I am concerned about the serious adverse effects of vaccines.
10. I do not need vaccines for diseases that are not common anymore.

### Statistical analysis

Descriptive statistics were used to characterize the study population. Continuous variables were reported as mean values and standard deviation (SD) and categorical variables were reported as frequencies.

To assess differences between the periods before and after the onset of COVID-19 pandemic, the Wilcoxon test was applied to all 10 VHS items. Based on previous studies evaluating the psychometric properties, internal consistency and validity of the VHS^25,26^, items L1-L4 and L6-L8 were grouped and scored to create Factor 1 (“lack of confidence”), while items L5 and L9 were scored to create Factor 2 (“risk perception”). Following Shapiro et al.[25], positively worded items were reverse-coded by subtracting their scores from 6 to ensure consistent interpretability. Items L5, L9, and L10, which are negatively worded, were kept in their original direction as negative sentences presented to participants. All items were also combined to generate a global vaccine hesitancy score before and after the pandemic. Higher scores on the total VHS or its subscales indicate greater hesitancy, lack of confidence, and perceived risk. Cronbach’s alpha indicated good internal consistency for both periods: before (0.82; 95%CI = 0.80–0.84) and after (0.86; 95%CI = 0.84–0.87) the onset of the pandemic.

To explore the association between Factors 1 and 2, socioeconomic characteristics and COVID-19 vaccination profile (hesitancy and self-reported number of doses), two bivariable and two multivariable linear regression models were fitted, one for each factor variation. The dependent variable was the difference in factor scores before and after the onset of the pandemic. In the bivariable analyses, categorical variables with two categories were compared using Student’s *t*-test, variables with more than two categories using one-way ANOVA, and continuous variables using Pearson’s correlation coefficient. Covariates with a p-value ≤0.20 in the bivariate analyses were included in the multivariable regression models. A stepwise selection procedure (both backward and forward) based on the Akaike Information Criterion (AIC) were used to determine the final model while controlling for potential confounders. Multicollinearity was assessed using the Variance Inflation Factor (VIF). The significance level for all hypothesis tests was set at 5% (α = 0.05). Analyses were conducted using R software version 4.4.1 (https://www.r-project.org/), with the following packages: *tidyverse*, *Cronbach*, *psych*, and *MASS*.

## Results

### Socioeconomic profile

A total of 514 residents fully completed the VHS scale. The mean age of participants was 46.7 years (SD = 17.4), with a mean of 10.4 years of education (SD = 10.7). The majority of the study population were women (61.7%), non-white individuals (69.8%), and not classified as low income (64.6%). Most participants had access to piped water (95.3%), and sanitary sewerage (52.7%); however, most did not have health insurance (69.8%) (Table 1).

**Table 1:** Socioeconomic characteristics, COVID-19 vaccine hesitancy, and number os doses received among participants.

Variables	Mean (SD)
Age (years)	46.7 (17.4)
Education (years)	10.4 (10.7)
Residents per household	3.1 (1.5)
Sex	n (%)
Female	317 (61.7)
Male	197 (38.3)
Race/Ethnicity	
Black/Mixed/Indigenous/Asian descent	359 (69.8)
White	155 (30.2)
Access to piped water	
No	24 (4.67)
Yes	490 (95.3)
Access to sanitary sewerage	
No	243 (47.3)
Yes	271 (52.7)
Access to health insurance	
No	359 (69.8)
Yes	155 (30.2)
Low income	
No	332 (64.6)
Yes	182 (35.4)
Self-reported COVID-19 vaccine hesitancy	
No	366 (71.2)
Yes	148 (28.8)
Number of COVID-19 vaccine doses	
None	21 (4.1)
1	24 (4.7)
2	101 (19.7)
≥3	329 (64.0)

### Self-reported COVID-19 vaccination and vaccine hesitancy

Most participants reported no hesitation regarding COVID-19 vaccination (71.2%). The mean number of COVID-19 vaccine doses received was 2.9 (SD = 1.1), and 64% of residents reported having received three or more doses (Table 1). The question on the number of vaccine doses was added after the pilot study, resulting in 7.6% missing data (39 participants) for this variable.

### Vaccine Hesitancy Scale (VHS)

**Fig. 1:**
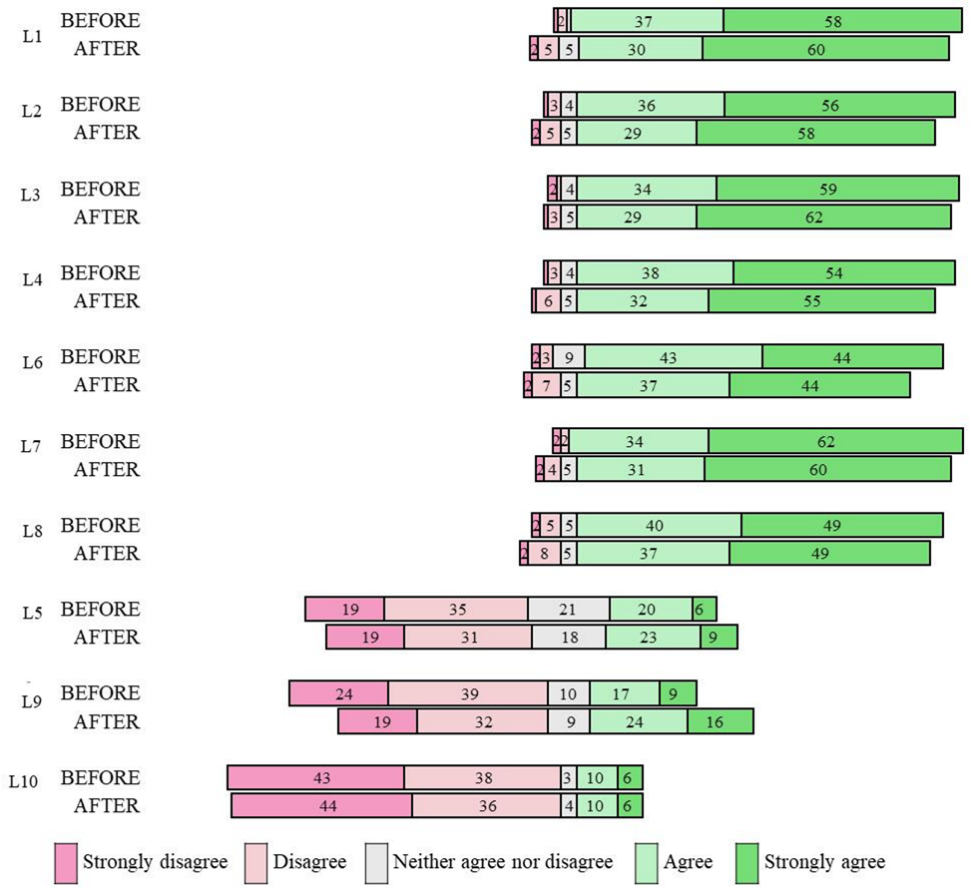
Distribution of responses to each Vaccine Hesitancy Scale (VHS) item, reflecting vaccine-related perceptions before and after the COVID-19 pandemic.

In the original (non-reversed) scale presented to participants, items L1–L4 and L6–L8 showed high levels of agreement Fig. 1. Items L5, L9, and L10 were associated with disagreement, with lower medians ranging from 2 to 3. Items L1 (p-value = 0.020), L4 (p-value = 0.015), L6 (p-value = 0.001), L7 (p-value = 0.003), and L8 (p-value = 0.014) showed a negative change between the pre- and post-pandemic periods, according to the Wilcoxon test, indicating reduced agreement (“agree” and “strongly agree”) and increased disagreement (“disagree” and “strongly disagree”). In contrast, items L5 and L9 showed a positive change, reflecting a higher level of agreement after the onset of the COVID-19 pandemic (p-value < 0.001) (Table 2).

**Table 2:** Median scores for each Vaccine Hesitancy Scale (VHS) item before and after the COVID-19 pandemic, and overall VHS score

Item	Median		Median difference	p-value*
	Before	After	Before – after difference (95%CI)	
L1: Vaccines are important for my health	5	5	-0.50 (-0.00 – -0.50)	0.020
L2: Vaccines are effective	5	5	-0.00(-0.50 – 0.00)	0.178
L3: Having myself vaccinated is important for the health of others in my community	5	5	0.00 (-0.00 – 0.50)	0.898
L4: All vaccines offered by the SUS in my community are beneficial	5	5	-0.50 (-0.00 – -0.50)	0.015
L5: New vaccines carry more risks than older vaccines	2	3	1.00 (0.50 – 1.00)	<.001
L6: The information I receive about vaccines from the vaccine program is reliable and trustworthy	4	4	-0.50 (-0.00 – -0.50)	0.001
L7: Getting vaccinated is a good way to protect myself from disease	5	5	-0.50 (-0.00 – -1.00)	0.003
L8: Generally, I do what my doctor or health care provider recommends about vaccines	4	4	-0.50 (-0.00 – -1.00)	0.014
L9: I am concerned about the serious adverse effects of vaccines	2	2	1.50 (1.00 – 1.50)	<.001
L10: I do not need vaccines for diseases that are not common anymore	2	2	-0.00 (-0.00 – 1.00)	0.430
VHS score (mean [SD])	18.2 [5.9]	19.2 [6.9]	0.96 (0.58 – 1.33)	<.001
Factor 1 (mean [SD])	11.2 (4.5)	11.6 (5.3)	0.40 (0.10 – 0.71)	0.008
Factor 2 (mean [SD])	5.7 (2.0)	5.6 (2.2)	0.53 (0.39 – 0.66)	<.001

*Median differences estimated using the Wilcoxon signed-rank test (Hodges–Lehmann estimator). Continuous scores compared using paired t-test.

After reversing positively worded items so that higher scores indicated greater hesitancy, the overall mean VHS score was 18.2 (SD = 5.9) before the COVID-19 pandemic and 19.2 (SD = 6.9) after its onset. The mean difference was 0.96 (95%CI = 0.58–1.33; p-value < 0.001), indicating increased vaccine hesitancy following the pandemic. For Factor 1 (lack of confidence), the mean score increased from 11.18 (SD = 4.49) pre-pandemic to 11.59 (SD = 5.32) post-pandemic (mean difference = 0.40; 95%CI = 0.10–0.71; p-value = 0.008), suggesting decreased confidence after the pandemic. For Factor 2 (risk perception), the mean score increased from 5.07 (SD = 2.00) to 5.60 (SD = 2.23) (mean difference = 0.53; 95%CI = 0.39–0.66; p-value < 0.001), reflecting greater perceived risk after the onset pandemic.

### Association between socioeconomic characteristics and VHS

In the bivariable analysis (Table 3), variables associated with Factor 1 included access to sanitary sewerage, access to health insurance coverage, self-reported COVID-19 vaccine hesitancy, and receipt of three or more COVID-19 vaccine doses. Variables with p-values ≤0.20 were age, years of education, access to sanitary sewerage, access to health insurance coverage, low income, self-reported COVID-19 vaccine hesitancy, and the number of COVID-19 vaccine doses received. In final adjusted exploratory model, significant associations were observed for age (ꞵ = 0.02; p-value = 0.005), self-reported COVID-19 vaccine hesitancy (ꞵ = 2.11; p-value < 0.001), and receipt three or more COVID-19 vaccine doses (ꞵ = −1.72; p-value = 0.024). Education, access to sanitary sewerage treatment, and health insurance coverage retained in the model for adjustment.

For Factor 2, the number of household residents, self-reported COVID-19 vaccine hesitancy, and number of vaccine doses received were associated with increased risk perception in the bivariable analysis. Variables with p-values ≤0.20 were the number of household residents, access to health insurance coverage, low income, COVID-19 self-reported vaccine hesitancy, and the number vaccine doses received. In the final adjusted model, self-reported COVID-19 vaccine hesitancy (ꞵ = 1.12; p-value < 0.001) remained significantly associated with increased perceived of risk, while number of household residents was retained for adjustment.

**Table 3:** Bivariable and multivariable analyses for Factor 1 (lack of confidence) and Factor 2 (risk perception).

Variables	Factor 1 (lack of confidence)						Factor 2 (risk perception)							
	Mean score (SD)		Bivariate analysis		Multivariable analysis		Mean score (SD)		Bivariate analysis			Multivariable analysis		
	Before	After	Pearson coef.	*p*	β [SE]	*p*	Before	After	Pearson coef.		*p*	β [SE]		*p*
Age	–	–	0.05	0.198	0.02 [0.01]	0.005	–	–	−0.02		0.516			
Education	–	–	0.06	0.116	0.02 [0.01]	0.067	–	–	0.02		0.536			
Household residents	–	–	−0.01	0.830			–	–	0.08		0.044	0.09 [0.04]		0.052
Sex			Mean variation [95%CI]						Mean variation [95%CI]					
Female	11.1 (4.5)	11.5 (5.2)	0.39 (−0.70–0.64)	0.932			5.01 (1.9)	5.53 (2.2)	0.52 (−0.30–0.25)		0.875			
Male	11.2 (4.5)	11.7 (5.6)	0.42 (−0.70–0.64)				5.18 (2.1)	5.72 (2.3)	0.54 (−0.30–0.25)					
Race/ethnicity														
Black/mixed/Indigenous/Asian descent	11.2 (4.7)	11.6 (5.4)	0.33 (−0.84–0.37)	0.446			5.1 (2.0)	5.7 (2.3)	0.58 (−0.10–0.45)		0.224			
White	11.0 (4.1)	11.6 (5.2)	0.57 (−0.84–0.37)				5.0 (2.0)	5.4 (2.2)	0.40 (−0.10–0.45)					
Access to piped water														
No	11.8 (3.5)	13.0 (5.2)	1.20 (−0.81–2.49)	0.307			5.6 (2.1)	5.8 (2.2)	0.25 (−1.20–0.61)		0.513			
Yes	11.1 (4.5)	11.5 (5.3)	0.36 (−0.81–2.49)				5.1 (2.0)	5.6 (2.2)	0.54 (−1.20–0.61)					
Access to sanitary sewerage														
No	11.7 (4.4)	11.7 (4.9)	0.02 (−1.33 – −0.13)	0.016	1	0.063	5.3 (2.0)	5.8 (2.2)	0.53 (−0.26–0.28)		0.937			
Yes	10.7 (4.5)	11.5 (5.6)	0.80 (−1.33 – −0.13)		0.58 [0.31]		5.0 (2.0)	5.4 (2.2)	0.52 (−0.26–0.28)					
Access to health insurance coverage														
No	11.5 (4.8)	11.7 (5.4)	0.19 (−1.42– −0.01)	0.046	1	0.111	5.1 (2.0)	5.7 (2.3)	0.60 (−0.03–0.51)		0.089			
Yes	10.4 (3.6)	11.3 (5.0)	0.90 (−1.42– −0.01)		0.55 [0.34]		5.0 (2.0)	5.4 (2.1)	0.36 (−0.03–0.51)					
Low income														
No	10.7 (4.4)	11.3 (5.4)	0.61 (−0.07– 1.23)	0.080			5.0 (1.9)	5.4 (2.2)	0.43 (−0.54–0.04)		0.094			
Yes	12.0 (4.6)	12.0 (5.2)	0.03 (−0.07– 1.23)				5.2 (2.1)	5.9 (2.2)	0.69 (−0.54–0.04)					
Self-reported COVID-19 vaccine hesitancy														
No	10.6 (4.0)	10.4 (4.0)	0.22 (−3.04– −1.38)	< 0.001	1		4.9 (1.9)	5.2 (2.1)	0.24 (−1.35 – −0.65)		< 0.001	1		< 0.001
Yes	12.6 (5.2)	14.6 (6.7)	2.00 (−3.04– −1.38)		2.11 [0.38]	< 0.001	5.5 (2.1)	6.7 (2.1)	1.24 (−1.35 – −0.65)			1.12 [0.15]		
COVID-19 vaccine doses														
None	17.0 (7.2)	19.0 (7.8)	−0.58 (−2.63–1.47)		−0.80 [1.00]	0.418	6.1 (2.0)	7.3 (1.7)	−0.34 (−1.27–0.57)					
1	13.0 (5.3)	15.6 (7.2)	2.58 (1.17–3.98)	< 0.001	1		5.1 (1.7)	6.8 (1.9)	0.44 (0.91–2.17)		< 0.001			
2	11.9 (4.5)	13.1 (5.9)	−1.46 (−3.02– 0.09)		−0.89 [0.77]	0.245	5.5 (1.9)	6.2 (2.1)	−0.48 (−1.56 – −0.16)					
≥3	10.3 (3.6)	10.4 (4.1)	−2.53 (−3.99 – −1.08)		−1.72 [0.75]	0.024	4.8 (2.0)	5.2 (2.2)	−0.44 (−1.83–0.53)					

## Discussion

The study’s descriptive analysis explored trends in vaccine hesitancy in the municipality of Campo Grande, located in Center-East Brazil, during the transitional phase of the pandemic’s conclusion, spanning the years 2022 and 2023.

The findings revealed a positive perception towards vaccination in the sample, both prior to and following the onset of the pandemic, reflected by higher scores in the confidence-related dimension of the VHS. This pattern is consisted with previous studies conducted in different populations and contexts, which have reported generally high levels of trust in vaccines and vaccination programs^24,26–29^. In the Brazilian context, this sustained confidence is likely influenced by the long-standing National Immunization Program (PNI), which has established a robust culture of immunization and consistently increased vaccine coverage over the years^30^.

Despite this overall positive perception, both VHS dimensions - “Lack of Confidence” and “Risk perception” - demonstrated a significant increase after the onset of the pandemic, indicating heightened vaccine hesitancy over time. These findings suggest that the COVID-19 pandemic negatively affected both confidence in vaccines and risk perception in the studied municipality in Brazil. While evidence from other studies varies depending on context and methodology, several investigations have reported changes in vaccine-related attitudes during the pandemic, including increased uptake of certain vaccines, such as influenza among adults^31^.

When results were analyzed at the aggregated level, both VHS dimensions demonstrated a significant post-pandemic shift toward greater vaccine hesitancy. The confidence dimension showed a marked decline, reflecting increased disagreement with statements related to the perceived benefits of vaccination, trust in vaccines offered by the public health system, reliability of information provided by vaccination programs, and adherence to health care providers’ recommendations. Concurrently, the risk perception dimension increased, indicating greater agreement with statements expressing concerns about the safety of newer vaccines and potential for serious adverse effects.

These specific changes, alongside the increase in those scores among participants who identified themselves as COVID-19-specific hesitant, underscored growing institutional distrust and decrease in confidence in healthcare workers, providers, and vaccines within the population. Although the present study did not directly assess exposure to misinformation or political influences, these findings may be contextualized in light of broader phenomena described in the literature. In particular, the COVID-19 infodemic that unfolded during the preceding years has been widely associated with increased mistrust and raised doubts about COVID-19 vaccines^28,29,34^. Additionally, political influences also negatively impacted vaccine uptake during the pandemic. In Brazil, for example, lower vaccination rates have been associated with the political stance and public discourse of the Federal President at the time, Jair Messias Bolsonaro^35^, illustrating the significant role that political influences and vaccine-related controversies may play in shaping public hesitancy^36^.

A higher number of COVID-19 vaccine doses was associated with increased confidence. This finding underscores the importance of vaccination as a social norm, as vaccine uptake within a social network can influence others to accept vaccination^32,37^. Moreover, the observed decrease in lack of confidence emphasizes the potential of using vaccine hesitancy as a predictor for vaccine uptake^33,38^. Lastly, in our study, higher age was associated with a higher lack of confidence. Older individuals have been previously correlated with greater risk assessment and concerns regarding side effects^39,40^.

Our study holds limitations, the most significant being its descriptive nature, as the assessment was conducted cross-sectionally rather than longitudinally. Our effect sizes were small, considering the low hesitancy assessed through the VHS. In order to enable comparability between before and after perceptions, we kept a raw score ranging from 5 to 50 overall. Anyhow, our study aimed to explore early shifts in perceptions about vaccines in the studied population, which may also represent shifts in attitudes toward vaccines. Additionally, since data collection occurred during the first year following the end of the health emergency declaration, perceptions of hesitancy may have been heightened given the novelty of the COVID-19 pandemic during that period. Besides, recall bias may be considered, as the items’ scores were assigned at one point in time, after the COVID-19 onset, regarding their past and present perceptions about vaccination.

## Conclusion

The results showed an increase in vaccine hesitancy, between the periods pre and post the COVID-19 pandemic, within the studied population, marked by a loss of confidence and a decrease in risk perception. The determinants associated with higher vaccine hesitancy scores included older age and hesitation to receive COVID-19 immunization, while a greater uptake of COVID-19 booster doses was linked to a lower change in hesitancy scores. These findings suggest a negative impact of the COVID-19 pandemic on the overall perception of vaccines in the population and highlighted the need for further studies to better comprehend the long-term effects of the pandemic on vaccine hesitancy.

## Supplementary Information

Below is the link to the electronic supplementary material.

Supplementary Material 1

## Data Availability

Data is provided within the manuscript or supplementary information files.
