# Supplementary material for: COVID-19 pandemic and vaccine hesitancy in a Brazilian state capital
Source: Sci Rep. 2026 Mar 25;16:14923. doi: 10.1038/s41598-026-45085-0 (PMC13168230; doi:10.1038/s41598-026-45085-0)
Supplement: Supplementary file 1 — Supplementary Material 1 [file 41598_2026_45085_MOESM1_ESM.docx]

**S1 Appendix**

The sampling method was conducted according to the cluster sampling proposed by the WHO, in 2018, for studies that aim to estimate vaccination coverage. This method is based on two stages: (1) cluster selection and (2) households selection.

1. Definition and selection of clusters: assuming that the expected average vaccination coverage in Campo Grande, for all vaccines available in the PNI, is 90%, with a confidence interval around the estimates of 8% (i.e., 90% ± 8% coverage estimate), with an alpha (type I error) of 5%, the effective sample size – based on an assumption of simple random sampling – was $n$ = 101 (Table 1).

**Table 1** Reproduction of Table B-1 – Effective sample size (ESS) by expected coverage and desired precision for the 95% confidence interval (CI)

| **Precision for 95% CI** | **Expected Coverage** | | | | | | | | | | |
| --- | --- | --- | --- | --- | --- | --- | --- | --- | --- | --- | --- |
|  | **5%** | **10%** | **15%** | **20%** | **25%** | **30 -70%** | **75%** | **80%** | **85%** | **90%** | **95%** |
| **±3%** | 354 | 518 | 663 | 788 | 892 | 1,097 | 892 | 788 | 663 | 518 | 354 |
| **±4%** | 227 | 315 | 394 | 461 | 517 | 622 | 517 | 461 | 394 | 315 | 227 |
| **±5%** | 162 | 216 | 265 | 306 | 340 | 401 | 340 | 306 | 265 | 216 | 162 |
| **±6%** | 132 | 160 | 192 | 220 | 242 | 280 | 242 | 220 | 192 | 160 | 132 |
| **±7%** | 110 | 125 | 147 | 167 | 182 | 207 | 182 | 167 | 147 | 125 | 110 |
| **±8%** | 93 | 101 | 117 | 131 | 143 | 159 | 143 | 131 | 117 | **101** | 93 |
| **±9%** | 81 | 83 | 96 | 106 | 115 | 126 | 115 | 106 | 96 | 83 | 81 |
| **±10%** | 70 | 70 | 80 | 88 | 95 | 103 | 95 | 88 | 80 | 70 | 70 |

Source: Annex B-1 of the WHO 2018 manual

To determine the average number of people eligible for the study (individuals aged 12 years or older), a pilot study was conducted. For this, one cluster (census tract) was randomly selected. Considering that on average, in Brazil, each census tract has approximately 300 households, for the pilot study 10% of the households contained in the selected cluster were drawn, which resulted in 31 households. After completing the pilot study, the average number of respondents per cluster, within a 3-hour interval, with a field team of 6 researchers, distributed in pairs, was 10. Assuming that the intracluster correlation is 0.33, the design effect size was set at 3.

| **Average Respondents per Cluster (m)** | | | | | | | |
| --- | --- | --- | --- | --- | --- | --- | --- |
| **ICC** | **1** | **5** | **7** | **10** | **15** | **20** | **Description** |
| **0** | 1 | 1 | 1 | 1 | 1 | 1 | Uniform coverage |
| **0.042** | 1 | 1.17 | 1.25 | 1.38 | 1.58 | 1.79 | ICC = 1/24; very little variation in coverage |
| **0.167** | 1 | 1.67 | 2 | 2.50 | 3.33 | 4.17 | ICC = 1/6; conservative choice for SIA surveys |
| **0.333** | 1 | 2.33 | 3 | 4 | 5.67 | 7.33 | ICC = 1/3; conservative choice for RI surveys |
| **1** | 1 | 5 | 7 | 10 | 15 | 20 | Some clusters 100% covered; all others 0% |

Applying the formula proposed by the WHO, the estimated number of clusters was 30.3, which was rounded to 30 (equation **a**). The selection of clusters was done by simple random sampling without replacement, using the cartographic base of census sectors from the IBGE of 2021. The cluster where the pilot study was conducted was included in the study. Therefore, an additional 29 clusters were sampled afterward. Clusters that primarily contained institutionalized populations (prisons and long-term care facilities, such as nursing homes) were immediately replaced when drawn. Clusters containing large condominiums or gated communities that did not allow the study team entry for data collection after initial contact by the researchers were also replaced (Figure 1).

1. $\boldsymbol{N}\text{clusters}\boldsymbol{=}\frac{\boldsymbol{ESS\times DEFF}}{\boldsymbol{m}}\boldsymbol{\to}\frac{\boldsymbol{101\times3}}{\boldsymbol{10}}\boldsymbol{\to30.3\to30*}$

**Table** Summary of terms of equations for sampling

| **Item** | **Equation term** | **Input** | | **Term value** |
| --- | --- | --- | --- | --- |
| a | Number of strata | No input | | 1 |
| b | Effective sample size estimation with desired precision (ESS) | Expected coverage | 90% | 101 |
|  |  | Precision level | 8% |  |
| c | Design effect | m (eligible participants per cluster) | 10 | 3 |
|  |  | ICC – Intracluster correlation coefficient | 0.33 |  |
|  |  | CV – coefficient of variation | 1.05 |  |
| d | Number of households to visit to find an eligible participant | No input | | 1.5 |
| e | Nonresponse | Eligible and not respond | | 1.05 |

**Source:** Adapted from WHO 2018 manual

**Figure 1** Selected clusters for the household survey.


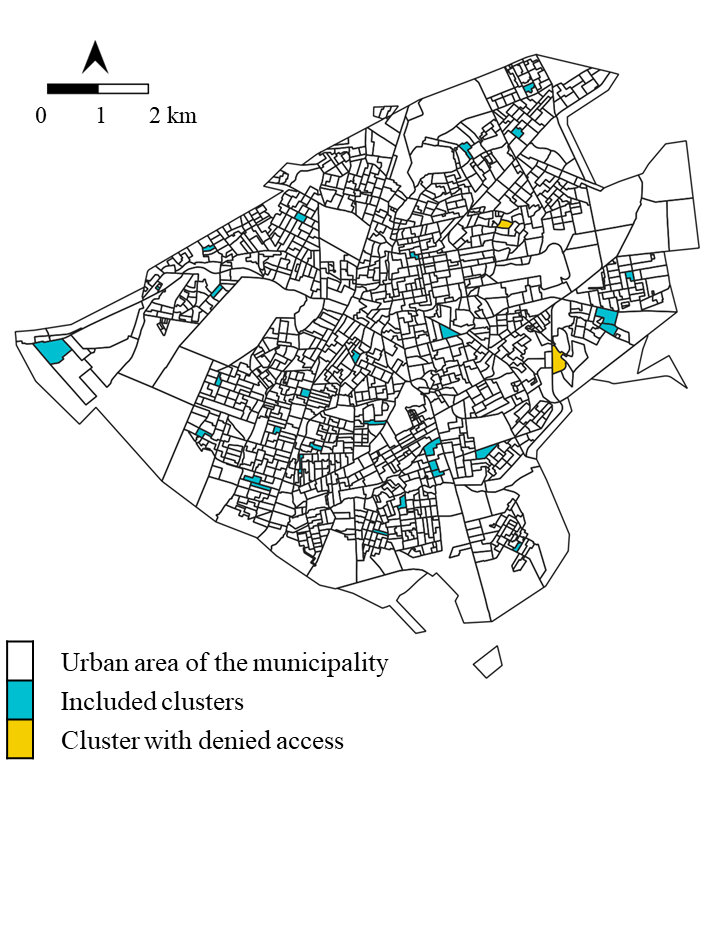


Source: Adapted from IBGE cartographic base of census sectors

1. The definition of the number and selection of the households was based on the pilot study. The number of residences visited to find an eligible participant averaged 1.5, the inflation factor to account for refusals and non-respondent residences was 1.05, and the average number of respondents per day of data collection was 10. Therefore, the number of households per cluster was defined as 15 (equation **b**).
2. $\boldsymbol{N}\text{holseholds to visit per cluster}\boldsymbol{=D \times E \times m \to1.5 \times1.05 \times10 = 15}$

Those households were selected by random simple probabilistic sampling. The random sampling and spatial allocation of clusters and households were performed using *sf* package from software R 3.4.2.

**References**

WAGAI, J. N.; RHODA, D.; PRIER, M.; TRIMMER, M. K.; CLARY, C. B.; OTERI, J.; OKPOSEN, B.; ADENIRAN, A.; DANOVARO-HOLLIDAY, C.; CUTTS, F. Implementing WHO guidance on conducting and analysing vaccination coverage cluster surveys: Two examples from Nigeria. **PLoS ONE**, v. 16, n. 2 Febuary, p. 1–24, 2021. Disponível em: <http://dx.doi.org/10.1371/journal.pone.0247415>.

WHO. **World Health Organization Vaccination Coverage Cluster Surveys: Reference ManualWorld Health Organization**2018. Disponível em: <http://www.who.int/immunization/documents/who_ivb_18.09/en/>.

IBGE. **Malha de Setores Censitários**. 2022. Disponível em: https://www.ibge.gov.br/geociencias/organizacao-do-territorio/estrutura-territorial/26565-malhas-de-setores-censitarios-divisoes-intramunicipais.html. .
